# Enhanced Family‐Centered Care and Family Integrated Care for Preterm Infants in Neonatal Intensive Care Units: Comparative Outcomes and Implementation Features—A Scoping Review

**DOI:** 10.1002/pdi3.70070

**Published:** 2026-09-25

**Authors:** Aditya Hemendra Bhatt, Somashekhar Marutirao Nimbalkar, Dipen Vasudev Patel, Reshma Kushal Pujara

**Affiliations:** ^1^ Department of Neonatology, Pramukhswami Medical College Bhaikaka University Karamsad Gujarat India

**Keywords:** family‐centered care/family integrated care (FCC/FICare), implementation effectiveness/evidence synthesis, neonatal intensive care units (NICU), neurodevelopmental outcomes, parental involvement/family engagement, parental psychosocial well‐being/maternal stress, preterm infants/premature neonates, scoping review

## Abstract

This scoping review maps and compares evidence on enhanced or modified Family‐Centered Care (FCC) models—including Family Integrated Care (FICare) and related variants—against standard FCC/usual neonatal intensive care unit (NICU) care for preterm infants. We aimed to summarize the comparative effects on neonatal growth and development, feeding and hospital utilization metrics, parental engagement, and parental psychosocial outcomes, and to describe program characteristics and contextual factors reported alongside variation in results across settings. We included diverse study designs conducted in NICUs across multiple regions. Growth‐related outcomes were the most commonly reported domain: 30 studies assessed weight gain, growth velocity, feeding progression, discharge weight, or breastfeeding, and 15 studies explicitly reported better growth or feeding‐related outcomes with enhanced FCC/FICare than with standard FCC/usual care. Developmental outcomes were reported in 12 studies, parental psychosocial outcomes in 25 studies, hospital utilization metrics in 28 studies, and parental engagement measures in 22 studies. Benefits were most often described in programs that combined structured parent education with active bedside caregiving, participation in rounds, mentoring, or other implementation supports, although findings varied by program intensity, unit context, and implementation fidelity. Overall, the evidence supports enhanced FCC/FICare as a promising strategy for improving infant–family outcomes in NICUs, while highlighting the need for clearer component definitions, standardized outcome reporting, and longer‐term follow‐up to support scalable, sustainable implementation.

## 1 Introduction

Comparing enhanced Family‐Centered Care (FCC) programs with standard FCC for preterm infants has become increasingly important because prematurity remains common worldwide and is associated with complex short‐ and long‐term consequences. Global estimates suggest preterm birth affects roughly one in 10 births, with meaningful regional variation (e.g., reported rates in Alberta, Canada) [1, 2]. Alongside advances in neonatal medicine, many neonatal intensive care units (NICUs) have shifted from clinician‐led models toward approaches that more explicitly incorporate parents as partners in care [3, 4]. This shift matters clinically and socially: prematurity is linked to higher risks of developmental and neurobehavioral impairments, while families may experience substantial psychological distress during prolonged NICU admission [1, 5]. These downstream effects place pressure on health systems and can disrupt family functioning well beyond discharge [6, 7, 8]. Although the principles of FCC are widely endorsed, their operational definitions and real‐world implementation vary across units, resulting in inconsistent levels of parental participation and mixed outcome signals [9, 10]. Enhanced models such as Family Integrated Care (FICare) were designed to address these gaps by positioning parents as active members of the care team and supporting them through structured education and supervised participation [11, 12]. However, important uncertainties remain about how much benefit enhanced FCC/FICare provides compared with standard FCC, and which components are most influential across different health systems and cultures [13, 14]. The literature reports both positive and heterogeneous findings—some studies describe improvements in growth or length of stay, whereas others report limited or variable effects on neurodevelopment and parental stress [4, 15, 16]. Clarifying these patterns is necessary to avoid underuse of effective family‐centered interventions and to guide NICUs toward models that improve both infant and caregiver outcomes [6, 17]. In this review, we map the comparative outcomes and implementation characteristics to provide a structured overview of what is known, where evidence is strongest, and which gaps should be prioritized in future research and practice [12, 18].

## 2 Materials and Methods

We conducted a scoping review to systematically map and characterize studies that compared enhanced or modified FCC models (including FICare variants) with standard FCC or usual care for preterm infants cared in NICUs. A scoping approach was selected because we anticipated heterogeneity in program components, study designs, and outcome definitions; accordingly, our objective was to describe the breadth and patterns of evidence and identify gaps rather than to generate a single pooled effect estimate. Reporting was guided by the Preferred Reporting Items for Systematic Reviews and Meta‐Analyses Extension for Scoping Reviews (PRISMA‐ScR).

### 2.1 Study Design and Registration

A protocol and screening framework were developed a priori to guide eligibility decisions, items to be extracted, and synthesis. The review was reported in line with PRISMA‐ScR recommendations, with methods chosen to maximize transparency and reproducibility given the diversity of included evidence.

### 2.2 Eligibility Criteria

Eligibility criteria were prespecified using the Population–Concept–Context (P–C–C) framework and applied consistently during screening and full‐text assessment. The criteria were as follows:

Population: Preterm infants (gestational age ≤ 37 weeks at birth, and birth weight was irrespective) admitted to a NICU.

Concept: FCC and enhanced/modified FCC models (including FICare, mFICare, Close Collaboration with Parents, mobile‐supported FCC, and related variants) where parents had a structured, supported role beyond standard visiting/participation norms.

Context: NICU care settings, without restriction by geography or health‐system type. Studies conducted in non‐NICU settings were excluded.

### 2.3 Information Sources and Search Strategy

We searched five electronic databases (PubMed/MEDLINE, Embase, CINAHL, Cochrane Library, and Scopus) using a strategy combining controlled vocabulary and free‐text terms for family‐centered care, family integrated care, NICU, and preterm infants. The full PubMed search strategy was as follows; (family integrated care OR FICare OR family centered care OR family‐centered care OR family‐centered nursing) AND (preterm OR premature OR very low birth weight OR VLBW OR low birth weight) AND (neonatal OR neonate OR NICU OR neonatal intensive care). Adapted versions of this strategy were used for each database.

Searches were run between August and October 2025. To reduce the risk of missing relevant evidence, we also performed forward and backward citation searching of key papers and screened reference lists of relevant reviews.

### 2.4 Study Selection Process

Study selection proceeded in three steps. First, titles and abstracts were screened for potential relevance. Second, full texts were assessed against the P–C–C criteria. Third, eligible studies were grouped to support both descriptive mapping and deeper appraisal of the most informative evidence. Two reviewers independently performed screening at each stage using a standardized form, and disagreements were resolved by discussion and consensus; where needed, a third reviewer adjudicated.

In the first phase (initial screening), database searches yielded 262 unique records after deduplication. Citation tracking identified an additional 41 records (backward: *n* = 28; forward: *n* = 13), resulting in 303 records for screening. Titles and abstracts were reviewed independently by two reviewers using the prespecified P–C–C criteria; because this was a scoping review, we used an intentionally inclusive approach to retain potentially relevant evidence for full‐text assessment.

In the second phase (full‐text review), full‐text articles for all records retained after screening (*n* = 303) were retrieved and assessed. Five records were excluded at full‐text stage because the available versions lacked sufficient methodological detail or outcome data to support extraction. Reasons for exclusion were documented.

In the third phase (study categorization), a total of 298 studies met eligibility criteria and formed the evidence base. To enable a focused appraisal while preserving breadth, 38 studies were classified as “highly relevant” because they reported primary outcome data across at least three outcome domains and were judged as having low or moderate risk of bias. The remaining 260 studies contributed primarily to descriptive mapping and contextual synthesis.

Disagreements between reviewers at all phases were resolved through discussion and consensus; unresolved disagreements (*n* = 3) were arbitrated by a third reviewer (senior methodologist).

### 2.5 Data Extraction and Charting

We developed a standardized extraction form a priori, piloted it on five included studies, and refined it before full extraction. Two reviewers extracted data independently, with cross‐checking for accuracy and completeness.

We extracted the following information from each study.

Study characteristics: first author, publication year, country/region, study design (e.g., randomized controlled trial [RCT], cluster RCT, quasi‐experimental study, cohort), sample size, and infant characteristics.

Intervention characteristics: FCC/FICare model name, core components (e.g., parent education curriculum, bedside caregiving participation, involvement in rounds, peer support, mobile tools), intensity and duration of delivery, staff training, and implementation support.

Comparator: description of standard FCC/usual care or alternative FCC model, including parent access, education practices, and routine engagement opportunities.

Outcomes: we charted outcomes across the following five prespecified domains as follows.

1. Neonatal clinical and growth outcomes (e.g., weight gain/growth velocity, discharge anthropometrics, feeding milestones).
2. Neonatal safety and morbidity outcomes (e.g., nosocomial infections, readmissions, adverse events).
3. Neurodevelopmental and neurobehavioral outcomes (e.g., Bayley Scales, GMDS, DENVER II, or comparable measures).
4. Parental psychosocial outcomes (e.g., stress, anxiety/depression, self‐efficacy).
5. Parental engagement and participation measures (e.g., time at bedside, participation in caregiving tasks, involvement in care planning/rounds).

Where reported, we extracted summary statistics (means/standard deviations [SDs] or medians/interquartile ranges [IQRs]), effect estimates, and adjusted analyses alongside measurement time points.

### 2.6 Risk of Bias Assessment

Methodological quality was appraised using design‐appropriate tools by two reviewers independently, with disagreements resolved by consensus.

For randomized trials, we used the revised Cochrane Risk of Bias tool for randomized trials (RoB 2), which is designed to assess potential bias across core domains including the randomization process, deviations from intended interventions, missing outcome data, outcome measurement, and selective reporting. For non‐randomized intervention studies, we used the Risk Of Bias In Non‐randomized Studies—of Interventions (ROBINS‐I), which is intended to evaluate bias arising from confounding, participant selection, intervention classification, deviations from intended interventions, missing data, outcome measurement, and selective reporting. These design‐specific tools were chosen so that methodological appraisal matched the underlying study design. Findings were used to support interpretation of the evidence rather than to exclude studies from the scoping map.

Although meta‐analysis can help quantify between‐study variability when interventions, comparators, outcomes, and time points are sufficiently comparable, we judged formal pooling to be inappropriate for this scoping review. The included literature differed markedly in FCC/FICare components, comparator definitions, outcome measures, and assessment windows, so any pooled summary estimate would have been difficult to interpret meaningfully. Because the purpose of this review was to map the breadth and pattern of evidence rather than generate a single summary effect, we summarized comparative findings narratively and highlighted patterns by outcome domain, program characteristics, and context.

## 3 Results

Neonatal growth outcomes were the most commonly reported clinical domain: 30 studies assessed weight gain, growth velocity, feeding progression, discharge weight, or breastfeeding (Figure S1). In the comparative evidence summarized in Table 1, 15 studies explicitly reported higher weight gain, faster feeding progression, improved breastfeeding, or better discharge weight with enhanced FCC/FICare than with standard FCC/usual care. One study reported no difference in weight gain in intention‐to‐treat analysis but benefit was found in per‐protocol analyses, while the remaining studies were mixed, variably measured, or not focused on primarily growth. Developmental outcomes were reported in 12 studies, parental psychosocial outcomes in 25 studies, hospital utilization metrics in 28 studies, and parental engagement measures in 22 studies. Table 1 therefore serves as the mapping of comparative outcomes alongside key implementation features. Across the engagement literature, higher‐intensity programs such as FICare, mobile‐enhanced FICare, and Close Collaboration with Parents commonly combined structured parent education, bedside caregiving participation, involvement in rounds, mentoring, and sometimes technology‐supported communication, and these models were the ones most often associated with greater parental presence and participation than standard FCC/usual care [35]. In contrast, studies in which the intervention was narrower—for example, discharge‐preparation programs or communication‐focused support—more often reported targeted gains such as discharge readiness, communication, or stress reduction rather than broad neonatal effects.

**TABLE 1:** Summary of comparative outcomes and parental‐engagement features.

Study	Neonatal growth outcomes	Developmental progress	Parental psychosocial well‐being	Hospital utilization metrics	Parental engagement measures
(Moe et al., 2022) [1]	Improved communication delay risk; no early motor effect	Mixed developmental domain effects; communication improved at 6–24 months	No significant maternal distress difference at discharge	Shorter discharge by 2.55 days; no readmission difference	Parent integration into care team; confidence increased
(Franck et al., 2019) [9]	Infant growth as primary outcome; feasibility of mobile app support	Secondary infant outcomes assessed; developmental impact exploratory	Parental stress, competence, self‐efficacy measured	Length of stay compared; infection rates monitored	Parent participation tracked via app and rounds
(Yu et al., 2017) [19]	Earlier feeding, greater weight gain in VLBW infants	Better neurobehavioral performance in intervention group	Parental motivation linked to infant outcomes	Earlier hospital discharge	Parental adherence to interventions emphasized
(Franck et al., 2022) [11]	No weight gain difference in intention‐to‐treat; per‐protocol showed benefits	Not primary focus; clinical outcomes emphasized	No parent‐related adverse events; stress not primary outcome	Lower nosocomial infection in mFICare; no LOS difference	Parent mentoring, rounds, and classes linked to outcomes
(Alferink et al., 2024) [20]	Scalability focus; neonatal outcomes to be compared	Developmental outcomes included in protocol	Family mental health assessed	Short‐term newborn outcomes compared	Family empowerment as primary caregiver emphasized
(Myttaraki and Gale, 2024) [21]	Greater weight gain at day 21 in FICare group	Secondary outcomes included breastfeeding frequency	Lower parental stress and anxiety in FICare group	No significant difference in other secondary outcomes	Parent integration in care supported
(Tiryaki et al., 2024) [22]	Higher discharge weight; improved breastfeeding status	Not primary focus; readiness for discharge assessed	Enhanced parental readiness for discharge	Not primary focus	FICare improved parental discharge preparedness
(Kubicka et al., 2023) [23]	Not primary focus; parental stress reduction noted	Not primary focus	Lower parental stress with increased participation	Not primary focus	Educational app use improved communication
(Mariani et al., 2024) [10]	Variety of FCC interventions; growth outcomes variably reported	Neurobehavioral/developmental outcomes frequently assessed	Parental mental health frequently measured	Length of stay variably reported	Diverse parental involvement strategies documented
(Schuler et al., 2024) [24]	No overall PMA discharge change; earlier discharge in low morbidity infants	Not primary focus	Parental satisfaction with discharge timing increased	Reduced PMA at discharge	Enhanced FCC increased parental satisfaction
(Ahlqvist‐Björkroth et al., 2025) [25]	Improved infant growth and shorter hospital stays reported	FCC practices improved; neurodevelopment benefits noted	Maternal depressive symptoms decreased long‐term	Length of stay shortened	Staff training enhanced parent collaboration
(Benzies et al., 2025) [15]	No association between care model and developmental delay risk	Developmental delay risk assessed at 18 months	Maternal parenting stress linked to developmental risk	Not primary focus	Maternal psychosocial factors considered
(Raghupathy et al., 2025) [16]	FCC improved motor and cognitive development at 24 months	Meta‐analysis showed motor and cognitive gains	Limited evidence on psychosocial outcomes	Not primary focus	Early FCC initiation emphasized
(Alsadaan et al., 2023) [26]	Significant improvements in cognitive, motor, language scores	Neurodevelopmental gains observed	Not primary focus	Reduced length of stay by 4.3 days	Integrated FCC and developmental care combined
(Chen and Dong, 2022) [13]	Shorter feeding times, higher weight gain, exclusive breastfeeding	Growth and development improved	Higher parental self‐efficacy and family functioning	Shorter hospital stay and lower readmission	Parents as primary caregivers in NICU care
(Ou et al., 2023) [27]	Increased weight gain velocity in extremely preterm infants	Not primary focus	Not primary focus	Not primary focus	FICare improved weight gain during hospitalization
(Benzies et al., 2020) [2]	Reduced hospital length of stay by 2.55 days	Not primary focus	No significant maternal psychosocial differences	No readmission or ED visit differences	Parent education and support emphasized
(Moreno‐Sanz et al., 2025) [12]	Improved short‐ and long‐term neonatal outcomes reported	Evolved to include critically ill infants	Psychological well‐being of families improved	Sustainability and scalability focus	Families empowered as primary caregivers
(North et al., 2022) [6]	Increased weight gain velocity; decreased ROP incidence	Neurobehavioral exam scores improved	Reduced parental stress and anxiety	Shorter hospital stay; lower infection rates	Direct parental bedside care included
(Zhang et al., 2018) [28]	Higher weight gain and breastfeeding rates	Not primary focus	Lower parental stress and higher satisfaction	Shorter NICU stay and reduced readmission	Parent education and participation promoted
(Yu et al., 2019) [29]	Enhanced neurophysiological maturation in VLBW infants	Improved neurobehavioral and neurophysiological function	Not primary focus	Not primary focus	Family‐centered intervention promoted parental adherence
(O'Brien et al., 2013) [3]	Improved weight gain and breastfeeding rates	Not primary focus	Reduced parental stress reported	Feasibility and safety confirmed	Parent education and mentoring included
(De Bernardo et al., 2017) [30]	Increased infant weight gain after 60 days	Not primary focus	Lower parental stress and higher satisfaction	Not primary focus	Expanded parental presence and active participation
(Itoshima et al., 2025) [31]	Shorter length of stay by 1.8 days; better growth rates	Not primary focus	Not primary focus	Reduced unscheduled outpatient visits	Unit‐wide FCC practice improvements
(Zhang et al., 2024) [14]	Not primary focus	Not primary focus	Significant reduction in maternal stress at discharge	Not primary focus	FICare reduced maternal stress in NICU
(Hannan and Bourque, 2020) [4]	Improved weight gain and breastfeeding rates	Not primary focus	Lower parental stress and anxiety	No mortality or major morbidity differences	Multilayer parental education and support
(Singh et al., 2024) [17]	Shorter hospital stay with high parental involvement	Not primary focus	Better parental mental health with higher involvement	Lower complication rates with high involvement	Parental participation frequency categorized
(O'Brien et al., 2015) [32]	Greater weight gain and breastfeeding rates	Not primary focus	Reduced parental stress and anxiety	Potential reduction in oxygen therapy duration	Parents as primary caregivers with staff support
(van Veenendaal et al., 2020) [33]	Reduced late‐onset sepsis and shorter hospital stay	Not primary focus	Not primary focus	Lower infection rates mediated by reduced catheter use	FICare in single family rooms emphasized
(Liu et al., 2022) [34]	Shorter length of stay; higher weight gain and feeding rates	Improved neuromotor and intellectual development	Lower parental anxiety and depression	Reduced nosocomial infection rates	Family participatory nursing enhanced engagement
(Lv et al., 2019) [7]	Higher discharge weight and improved nutrition outcomes	Not primary focus	Not primary focus	Reduced complications and readmission rates	Parental education and active care participation

Abbreviations: FCC, family‐centered care; PMA, postmenstrual age; VLBW, very low birth weight.

Overall, study quality varied and was closely linked to the transparency of intervention descriptions, handling of confounding factors, and completeness of outcome reporting. This variability limits the strength of causal inference in portions of the evidence base and underscores the need for clearer reporting of both implementation and outcomes.

Context appeared to matter. For example, unit‐wide models with formal staff training, peer support, and parents functioning as primary caregivers more often reported improvements in weight gain, breastfeeding, parental stress, or length of stay, whereas the multisite mFICare study reported no difference in weight gain in the intention‐to‐treat analysis but showed benefit mainly in per‐protocol analyses, suggesting that program uptake and fidelity influenced effect size. Similarly, zero‐separation or single‐family‐room models and Close Collaboration with Parents approaches appeared better able to support continuous parental presence and were linked in some studies to lower maternal stress, better growth, or shorter stay. In contrast, studies from resource‐constrained or space‐limited settings more often highlighted staffing constraints, infrastructure limitations, and inconsistent program fidelity as barriers that could narrow or attenuate outcome gains.

Taken together, the mapped literature supports this premise because the clearest signals clustered in domains closest to parent participation. Across 22 studies assessing parental engagement, greater parental presence or caregiving participation was the most consistent finding; across 25 studies assessing parental psychosocial outcomes, improvements related to stress, anxiety, confidence, self‐efficacy, or readiness for discharge were reported more often than null findings; and among the 30 growth‐related studies, 15 explicitly reported accompanying gains in feeding progression, weight gain, breastfeeding, or discharge weight. At the same time, effects were not uniform across all outcomes or settings, particularly for longer‐term developmental measures and for hospital metrics in studies where program intensity, fidelity, or available unit resources differed.

## 4 Conclusion

Enhanced FCC approaches—illustrated by FICare and related variants—are supported by a broad and diverse literature base and, in the mapped comparative evidence, were most consistently associated with better parental engagement, lower parental stress, and selected growth or feeding benefits. However, heterogeneity in program components, outcome measurement, and implementation fidelity complicates cross‐setting comparisons. Future work should define core components more clearly, standardize outcome sets (including longer‐term developmental follow‐up), and report implementation processes and fidelity to support scalable, sustainable adoption across diverse NICU contexts.

## Author Contributions

**Aditya Hemendra Bhatt:** conceptualized the review, developed the protocol, designed the search strategy, led study selection, data charting, and drafted the manuscript. **Somashekhar Marutirao Nimbalkar:** independently screened titles and abstracts, verified data extraction, and contributed to risk‐of‐bias assessments and manuscript revisions. **Dipen Vasudev Patel and Reshma Kushal Pujara:** provided clinical and methodological oversight, contributed to the interpretation of findings, and critically revised the manuscript for important intellectual content. All authors approved the final manuscript as submitting and agree to be accountable for all aspects of the work.

## Funding

The authors have nothing to report.

## Conflicts of Interest

The authors declare no conflicts of interest.

## Supporting information


**Figure S1:** PRISMA‐ScR flow diagram for study selection.

## Data Availability

No new data were generated or analyzed in this study. Data sharing does not apply to this article.
